# A Biomarker‐Driven and Interpretable Machine Learning Model for Diagnosing Diabetes Mellitus

**DOI:** 10.1002/fsn3.70234

**Published:** 2025-04-30

**Authors:** Zhihui Xiao, Mingfu Wang, Yueliang Zhao, Hui Wang

**Affiliations:** ^1^ College of Food Science and Technology Shanghai Ocean University Shanghai China; ^2^ Shenzhen Key Laboratory of Food Nutrition and Health, College of Chemistry and Environmental Engineering Shenzhen University Shenzhen China; ^3^ School of Public Health Shanghai Jiao Tong University School of Medicine Shanghai China

**Keywords:** biomarker‐driven, diabetes mellitus, interpretable, machine learning, prediction model

## Abstract

Diabetes is one of the leading causes of death and disability worldwide. Developing earlier and more accurate diagnosis methods is crucial for clinical prevention and treatment of diabetes. Here, data on biochemical indicators and physiological characteristics of 4335 participants from the National Health and Nutrition Examination Survey (NHANES) database from 2017 to 2020 were collected. After data preprocessing, the dataset was randomly divided into a training set (70%) and a test set (30%); then the Boruta algorithm was used to screen feature indicators on the training set. Next, three machine learning algorithms, including Random Forest (RF), Multi‐Layer Perceptron (MLP), and Extreme Gradient Boosting (XGBoost) were employed to build predictive models through 10‐fold cross‐validation on the training dataset, followed by performance evaluation on the test dataset. The RF model exhibited the best performance, with an area under the curve (AUC) of 0.958 (95% CI: 0.943–0.973), a recall of 0.897, a specificity and F1 score of 0.916 and 0.747, respectively, and an overall accuracy of 0.913. Moreover, SHapley Additive exPlanations (SHAP) and Partial Dependency Plots (PDP) were applied to interpret the RF model to analyze the risk factors for diabetes. Glycohemoglobin, glucose, fasting glucose, age, cholesterol, osmolality, BMI, blood urea nitrogen, and insulin were found to exert the greatest influence on the prevalence of diabetes. Collectively, the RF model has considerable application prospects for the diagnosis of diabetes and can serve as a valuable supplementary tool for clinical diagnosis and risk assessment in diabetes.

## Graphical abstract

Three machine learning algorithms were used to construct diabetes predictive models, among which the RF model had the best predictive ability, with an area under the curve (AUC) of 0.958 (95% CI: 0.943–0.973) and a recall of 0.897. SHAP and PDP methods were used to interpret the RF model and further analyze the risk factors for diabetes. Glycohemoglobin, glucose, fasting glucose, age, cholesterol, osmolality, BMI, blood urea nitrogen, and insulin were found to exert the greatest influence on the prevalence of diabetes. Collectively, the RF model has considerable application prospects for the identification of diabetes and can serve as a valuable supplementary tool for clinical diagnosis and risk assessment in diabetes.

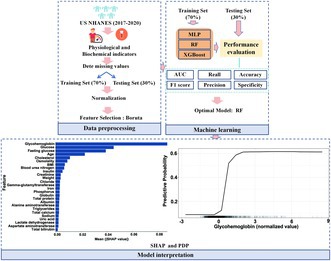

## 1 Introduction

Diabetes mellitus is a chronic metabolic disorder characterized by persistent hyperglycemia resulting from defects in insulin secretion, insulin action, or both (Yameny 2024). Currently, there are 463 million people with diabetes worldwide, and this number is expected to reach 783 million by 2045, with an estimated growth rate of 69.11% (Sugandh et al. 2023; Saeedi et al. 2019). The hyperglycemia caused by diabetes can damage the blood vessels of the heart, eyes, kidneys, and nerves and is associated with long‐term damage, dysfunction, and failure of organs (Tomic et al. 2022; Kopitar et al. 2020). Diabetes has been one of the leading causes of death and disability worldwide. For example, the risk of mortality from cardiovascular disease in patients with diabetes is 2 to 4 times higher than that in non‐diabetic patients (Gaye et al. 2024). Therefore, it is necessary to develop earlier and more accurate diagnostic methods for clinical prevention and treatment of diabetes.

Currently, diagnostic models based on machine learning technology have been constructed and applied to diagnose diseases such as chronic kidney disease, osteoporosis, coronary heart disease, and to analyze the risk factors that cause diseases (Islam et al. 2023; Tu et al. 2024; Ma et al. 2024; Husnain et al. 2024). For diabetes diagnosis, the physiological characteristics or biochemical indicators of diabetic patients have been collected for constructing diabetes diagnostic models using the CatBoost gradient boosting algorithm, logistic regression algorithm, naive Bayes algorithm, improved gray wolf optimization algorithm, and support vector machine algorithm. However, the area under the curve (AUC) (one of the important indicators for measuring model performance) values of these diagnostic models is around 0.8, and the accuracy rate is also below 0.85, which needs further improvement before clinical application (Kumar et al. 2022; Wu et al. 2018; Al‐Nussairi and Eljinini 2022; Zeinalnezhad and Shishehchi 2024). This may be because the clinical data sets of diabetes are usually highly complex, covering multi‐dimensional information such as diverse biochemical parameters, clinical manifestation, and pathophysiological changes. There may be complex interactions between this information and show nonlinear relationships, which limits the diagnostic performance of these algorithms. Therefore, it is necessary to use algorithms that can efficiently process nonlinear and highly complex data to build diabetes diagnostic models to improve the model's diagnostic performance.

The Random Forest (RF), Multilayer Perceptron (MLP), and Extreme Gradient Boosting (XGBoost) algorithms have powerful integrated learning, gradient boosting optimization, and deep nonlinear mapping capabilities. The RF algorithm and XGBoost algorithm have been found to diagnose heart disease effectively, with an AUC of 0.95 for both algorithms (Bhatt et al. 2023). The accuracy of diagnosing breast cancer using the MLP algorithm reached 91.32% (Sultana et al. 2021). We hypothesized that these three algorithms could diagnose diabetes more accurately.

While pursuing high‐precision diagnostic performance, model interpretation is equally important because it can reveal the logic behind the prediction and enhance the transparency and credibility of the decision‐making process. Currently, most machine learning algorithms use black box models, whose internal logic and decision‐making processes are complex and opaque. As a result, the constructed diabetes diagnosis models have poor interpretability and cannot analyze how each feature affects the model's prediction results, thus affecting our understanding of risk factors for diabetes prevalence (Lin et al. 2024). In terms of model interpretation methods, SHapley Additive exPlanations (SHAP) and Partial Dependency Plots (PDP) can decompose the model's diagnosis results into the influence of each feature, which enables us to better understand the model's decision‐making process and identify key features (Li 2022). Therefore, we used SHAP and PDP to interpret the model to help us analyze the risk factors for diabetes prevalence.

In this study, the physiological characteristics and biochemical indicators from a population cohort in the NHANES database in the United States, spanning 2017 to 2020, were collected, and three machine learning approaches: RF, MLP, and XGBoost, were employed to develop diagnostic models for diagnosing diabetic patients. SHAP and PDP were applied to interpret the model with the best diagnostic performance to analyze the risk factors for diabetes prevalence. The diabetes diagnostic model provided by this study can serve as a valuable supplementary tool for clinical diagnosis and risk assessment of diabetes.

## 2 Methods

### 2.1 Study Population

**FIGURE 1:**
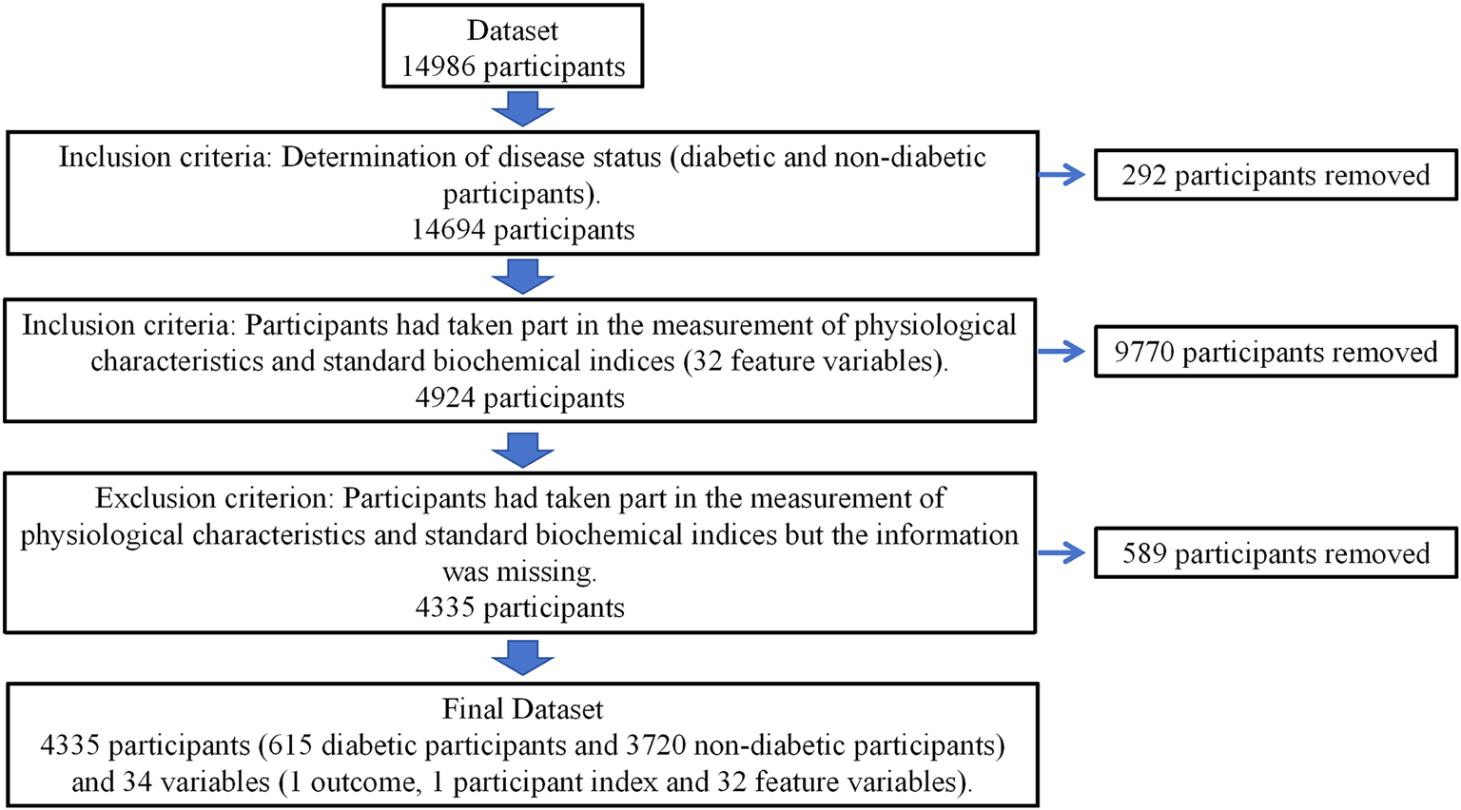
Flow diagram of data collection.

The data were obtained from the NHANES database (https://wwwn.cdc.gov/nchs/nhanes) in the United States, covering the period from 2017 to 2020. The inclusion criteria were as follows: (1) Participants' diabetic status information was confirmed according to NHANES health questionnaire data (DIQ010—Doctor told you have diabetes). (2) Participants had taken part in the measurement of physiological characteristics and standard biochemical indices. The exclusion criteria were as follows: Participants had taken part in the measurement of physiological characteristics and standard biochemical indices, but the information was missing. Finally, a total of 4335 participants were included in the final dataset, with 615 participants with diabetic status and 3720 participants with non‐diabetic status (Figure 1).

### 2.2 Data Collection

The physiological characteristics of participants were collected from questionnaire data in the NHANES database. Characteristics included gender, age (years), weight (kg), height (cm), and body mass index (BMI) (kg/m^2^). The biochemical indices of participants were collected from the standard biochemical index report. Twenty‐seven biochemical indices in blood were included in the final dataset, with all details supplied in Table 1.

**TABLE 1:** Biochemical indicators and physiological characteristics of the study population.

Variable	With diabetes					Without diabetes					*p*
	Mean	SD	Lower quantile	median	Upper quantile	Mean	SD	Lower quantile	Median	Upper quantile	
Gender	—	—	—	—	—	—	—	—	—	—	—
Age (years)	61.74	13.14	54.00	63.00	71.50	42.42	20.14	24.00	41.00	59.00	< 0.001
Weight (kg)	91.14	25.19	73.40	87.30	104.45	79.61	22.68	63.60	76.40	91.60	< 0.001
Height (cm)	166.04	9.81	159.00	165.30	173.20	166.65	10.02	159.20	166.30	173.80	0.189
Body mass index (BMI) (kg/m^2^)	32.88	7.91	27.60	31.40	36.90	28.57	7.49	23.50	27.30	32.20	< 0.001
Glycohemoglobin (%)	7.53	1.82	6.30	7.00	8.20	5.49	0.55	5.20	5.40	5.70	< 0.001
Insulin (μU/mL)	25.73	47.32	8.30	13.39	24.19	13.11	13.35	6.15	9.74	15.62	< 0.001
Fasting glucose (mg/dL)	161.26	64.19	118.00	142.00	180.50	102.63	18.38	94.00	100.00	107.00	< 0.001
Alanine aminotransferase (U/L)	23.02	16.06	14.00	18.00	26.50	21.11	20.32	12.00	16.00	24.00	< 0.001
Albumin (g/dL)	3.92	0.36	3.70	3.90	4.10	4.06	0.34	3.90	4.10	4.30	< 0.001
Alkaline phosphatase (IU/L)	86.37	36.33	66.00	80.00	98.00	88.50	52.00	62.00	76.00	95.00	0.003
Aspartate aminotransferase (U/L)	21.65	14.77	15.00	18.00	23.00	21.62	15.26	16.00	19.00	23.00	0.156
Bicarbonate (mmol/L)	25.51	2.55	24.00	26.00	27.00	25.44	2.34	24.00	25.00	27.00	0.396
Blood urea nitrogen (mg/dL)	17.95	8.88	12.00	16.00	21.00	13.71	4.93	10.00	13.00	16.00	< 0.001
Chloride (mmol/L)	100.62	3.32	99.00	101.00	103.00	101.88	2.54	100.00	102.00	104.00	< 0.001
Creatine phosphokinase (IU/L)	139.47	138.89	68.50	98.00	161.50	174.47	440.14	75.00	112.00	179.00	< 0.001
Creatinine (mg/dL)	1.05	1.07	0.69	0.87	1.10	0.84	0.31	0.68	0.81	0.95	< 0.001
Globulin (g/dL)	3.18	0.49	2.80	3.10	3.50	3.10	0.43	2.80	3.10	3.40	0.001
Glucose (mg/dL)	151.92	60.92	111.00	134.00	170.00	95.39	17.52	87.00	93.00	100.00	< 0.001
Gamma‐glutamyltransferase (IU/L)	44.43	109.11	17.50	25.00	43.00	27.53	35.29	13.00	19.00	28.00	< 0.001
Iron (μg/dL)	81.95	31.32	60.00	80.00	101.00	90.20	37.86	64.00	86.00	111.25	< 0.001
Lactate dehydrogenase (IU/L)	161.28	50.03	135.00	154.00	178.00	158.02	34.96	136.00	154.00	174.00	0.588
Osmolality (mmol/Kg)	284.95	6.41	281.00	285.00	289.00	281.02	5.03	278.00	281.00	284.00	< 0.001
Phosphorus (mg/dL)	3.56	0.58	3.20	3.50	3.90	3.59	0.58	3.20	3.60	3.90	0.306
Potassium (mmol/L)	4.18	0.43	3.90	4.10	4.40	4.11	0.34	3.90	4.10	4.30	0.001
Sodium (mmol/L)	140.39	2.95	138.00	141.00	142.00	140.77	2.42	139.00	141.00	142.00	0.003
Total bilirubin (mg/dL)	0.49	0.29	0.30	0.40	0.60	0.49	0.30	0.30	0.40	0.60	0.887
Total Calcium (mg/dL)	9.27	0.43	9.00	9.30	9.50	9.26	0.36	9.00	9.30	9.50	0.508
Cholesterol (mg/dL)	169.71	42.67	140.00	164.00	191.50	181.16	40.48	153.00	177.00	205.00	< 0.001
Total protein (g/dL)	7.09	0.49	6.80	7.10	7.40	7.16	0.43	6.90	7.20	7.40	< 0.001
Triglycerides (mg/dL)	147.39	90.92	94.00	128.00	177.00	110.96	67.80	67.00	93.00	133.25	< 0.001
Uric acid (mg/dL)	5.70	1.58	4.50	5.50	6.60	5.33	1.40	4.30	5.20	6.20	< 0.001

*Note:* Wilcoxon two‐sample test was used, and *p* < 0.05 was considered statistically significant.

### 2.3 Data Preprocessing and Feature Selection

To assess the diagnostic performance of the machine learning models in new unseen data, we randomly divided the dataset into a training set (70%) and a test set (30%) using stratified sampling. In the diabetes dataset, the units and value ranges of different features (such as blood sugar, age, BMI, etc.) vary greatly. If the original data is used directly to build the model, the model training will over‐focus on features with large value ranges and ignore features with small value ranges, which will reduce the diagnostic accuracy. *Z*‐score normalization puts each feature at the same level, thereby avoiding the impact of dimensional differences on the model and helping to improve the prediction accuracy of the model. So, we used the *Z*‐score standardization method on the training set to eliminate the impact of these differences. The *Z*‐score standardization formula is as below:

$$X\mathrm{norm}=\frac{x-\mu }{\sigma }$$

In this formula, *X*norm represented the normalized data, *x* the original data, *μ* represented the mean of the feature, and σ represented the standard deviation of the feature. Further, the *μ* and the *σ* obtained from the training set were applied to the test set.

Since the characteristics of the diabetes dataset are complex, there may be features that are not related to diabetes, which will interfere with the model training process and cause the model's diagnostic performance to deteriorate. So, we used the Boruta algorithm to perform feature selection in the training set, and then apply the selected features to the construction of the machine learning model. The process of feature selection using the Boruta algorithm is mainly implemented using R and the Boruta package. The parameter settings of the Boruta algorithm are *n*_Trees_ = 500, maxRuns = 500, and *p* values = 0.01.

### 2.4 Construction of the Model

Three machine learning methods, including RF, MLP, and XGBoost, were employed to construct the diagnostic model based on the 10‐fold cross‐validation method. RF is a supervised learning algorithm based on ensemble learning, which is widely used in classification and regression tasks. It improves the accuracy and stability of the model by building multiple decision trees and integrating their diagnostic results (usually voting or averaging). MLP is a basic feed‐forward neural network. It consists of an input layer, a hidden layer, and an output layer, with weights connecting the layers. By learning linear and nonlinear relationships in data, an MLP can quickly classify or predict outcomes. XGBoost is a powerful machine learning algorithm based on the gradient boosting framework, which builds a strong diagnostic model by combining multiple weak learners (decision trees). It efficiently processes large‐scale data, supports tasks such as classification and regression, and is widely used in many fields, known for its high performance and accuracy.

Construction of the models is implemented using R, the randomForest package, nnet package, and xgboost package. The grid search method was used for parameter tuning. The grid tuning parameters of RF were mtry, ntree, and min_n. The grid tuning parameters of MLP were hidden units and penalty. The grid tuning parameters of XGBoost were mtry, min_n, tree_depth, learn_rate, and loss_reduction.

### 2.5 Model Evaluation and Interpretation

The evaluation of the diagnostic model performance was conducted utilizing the following metrics: AUC (95% CI), accuracy, sensitivity/recall, specificity, F1 score, and precision.

In order to improve the interpretability of our model and explore the risk factors affecting diabetes, the machine learning interpretable tools SHAP and PDP were used for model interpretation. SHAP is mainly implemented using the fastshap package. PDP is mainly implemented using the iml package.

### 2.6 Statistical Analysis

Continuous variables in this study were described in the form of means and standard deviations. Comparisons between groups were made using the Wilcoxon two‐sample test with *α* = 0.05 as the test level, and *p* < 0.05 was considered statistically significant. The ggplot2 package of R was used for visualization. All statistical analyses were performed in R, version 4.2.1.

## 3 Results

### 3.1 Biochemical Indicators and Physiological Characteristics of the Study Population

A total of 4335 participants were included in this study, including 615 diabetic participants and 3720 non‐diabetic participants, with a diabetes ratio of 16.53%. The average weight of diabetic patients was 91.14 kg and the average BMI was 32.88 kg/m^2^, both of which were significantly higher than those of participants without diabetes (*p* < 0.05) (Table 1). Moreover, except for alkaline phosphatase, aspartate aminotransferase, bicarbonate, lactate dehydrogenase, phosphorus, total bilirubin, and calcium, the other biochemical indices of diabetic patients were significantly different from those without diabetes (*p <* 0.05) (Table 1). Overall, weight, BMI, and most biochemical indices were significantly different between diabetic and non‐diabetic patients, suggesting that these indices may be important indicators for distinguishing diabetic patients from those without diabetes.

### 3.2 Feature Selection

Diseases are often caused by multiple factors. To find out the factors most related to the occurrence of the disease, feature selection was performed. Some feature selection methods such as variance filtering and correlation filtering only consider the correlation between a single feature and the disease, so the accuracy of using the features obtained by these methods to diagnose disease outcomes is relatively low. The Boruta algorithm, which considers the interaction between features, was used for feature screening in this study. The feature screening results based on the Boruta algorithm were shown in Figure 1. After 500 iterations, 26 feature variables closely associated with diabetes were identified (*p* < 0.01), including age (years), weight (kg), body mass index(BMI) (kg/m^2^), glycohemoglobin (%), insulin (μU/mL), fasting glucose (mg/dL), alanine aminotransferase (U/L), albumin (g/dL), aspartate aminotransferase (U/L), blood urea nitrogen (mg/dL), chloride (mmol/L), creatinine (mg/dL), globulin (g/dL), glucose (mg/dL), gamma‐glutamyltransferase (IU/L), iron (μg/dL), lactate dehydrogenase (IU/L), osmolality (mmol/kg), phosphorus (mg/dL), sodium (mmol/L), total bilirubin (mg/dL), total calcium (mg/dL), cholesterol (mg/dL), total protein (g/dL), triglycerides (mg/dL), and uric acid (mg/dL). The 26 features selected were the most suitable feature variables for model construction to improve the model predictive performance.

### 3.3 Comparison of Model Performance

Different evaluation indicators can reflect the diagnostic performance and characteristics of the model from different angles. In our study, the primary metric for assessing the model's performance were AUC (95% CI) and Recall. AUC is not affected by the sample ratio and can stably evaluate the model's diagnostic performance. The larger the value represents the better the model's diagnostic performance. The Recall can directly reflect the model's ability to identify diabetic patients. The higher the Recall represent the stronger the model's ability to identify diabetic patients, thereby reducing the undetected rate. Secondary performance metrics were accuracy, specificity, F1 score, and precision.

**FIGURE 2:**
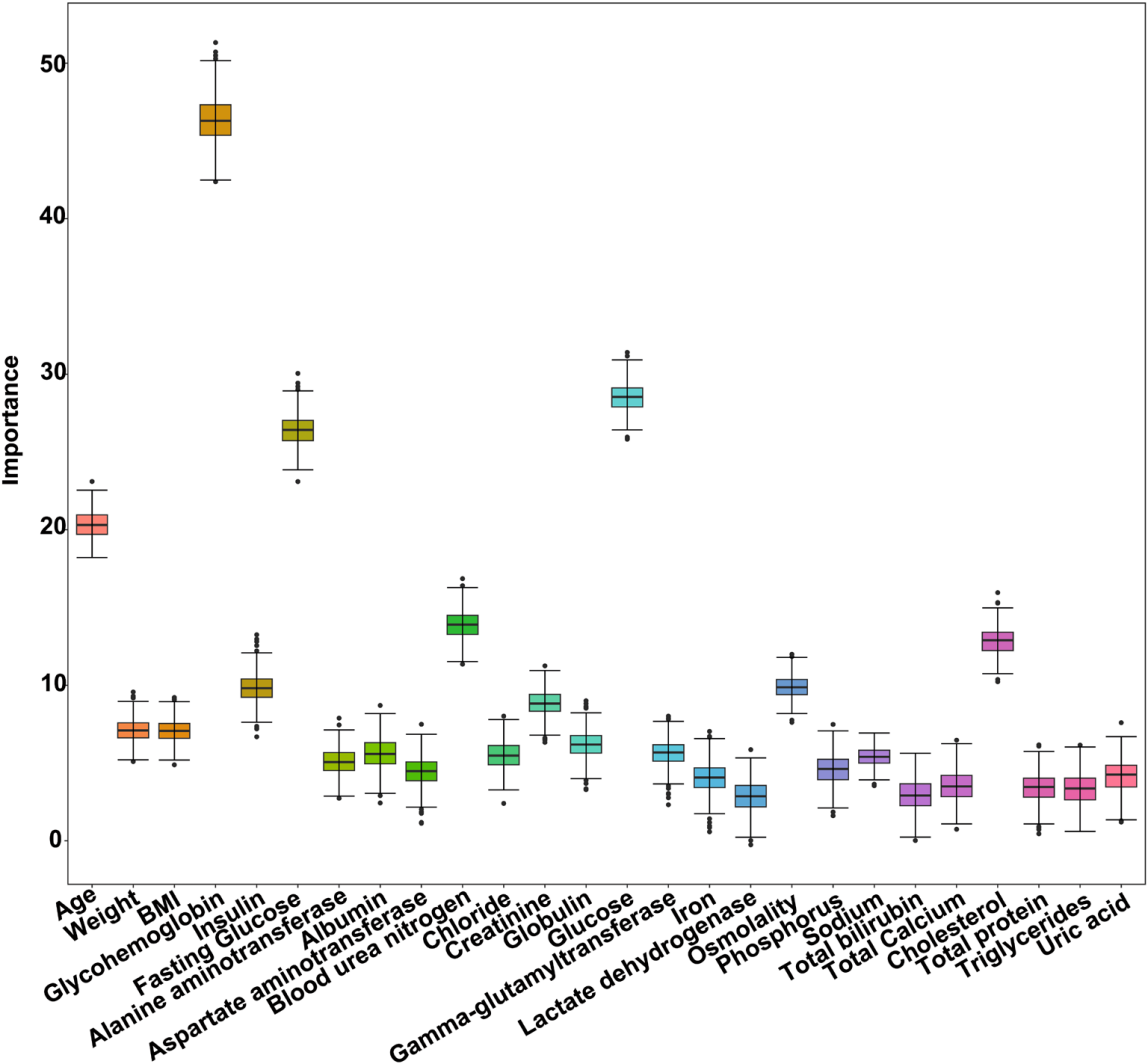
The screening of biomarkers for the diabetes prediction model based on the Boruta algorithm. After the Boruta operation, all features are divided into three categories: tentative, confirmed, and rejected. The features in the confirmed class were selected for modeling. The figure shows the importance scores of the features in the confirmed class.

RF model showed the best diagnostic ability among the three machine learning models, with an AUC of 0.958 (95% CI: 0.943–0.973), a recall of 0.897, a specificity of 0.916 and an F1 score of 0.747, and an overall accuracy of 0.913. The MLP model achieved an AUC of 0.956 (95% CI: 0.939–0.971), a recall of 0.859, a specificity of 0.923 and an F1 score of 0.741, and an overall accuracy of 0.914. The XGBoost model achieved an AUC of 0.955 (95% CI: 0.938–0.971), a recall of 0.881, a specificity of 0.919 and an F1 score of 0.744, and an overall accuracy of 0.913 (Figure 2 and Table 2). The AUC and recall of the RF diagnostic model were better than those of the MLP and XGboost diagnostic models. Therefore, the RF diagnostic model was selected for model interpretation to further explore the risk factors affecting diabetes.

**TABLE 2:** Comparison of the performance of three machine learning predictive model.

Algorithm	Sensitivity/recall	Specificity	F1 score	Precision	Accuracy
MLP	0.859	0.923	0.741	0.651	0.914
XGBoost	0.881	0.919	0.744	0.644	0.913
RF	0.897	0.916	0.747	0.640	0.913

### 3.4 Model Interpretation

Model interpretation allows us to delve deeper and analyze the multiple factors that influence disease risk. SHAP and PDP are both methods used to improve model interpretability. SHAP calculates the marginal contribution of each feature to the model's predicted output, thereby determining how much each feature affects the prediction. This helps understand the model's diagnostic logic, thereby improving the model's interpretability and transparency. PDP can show how changes in features affect the model's diagnosis.

**FIGURE 3:**
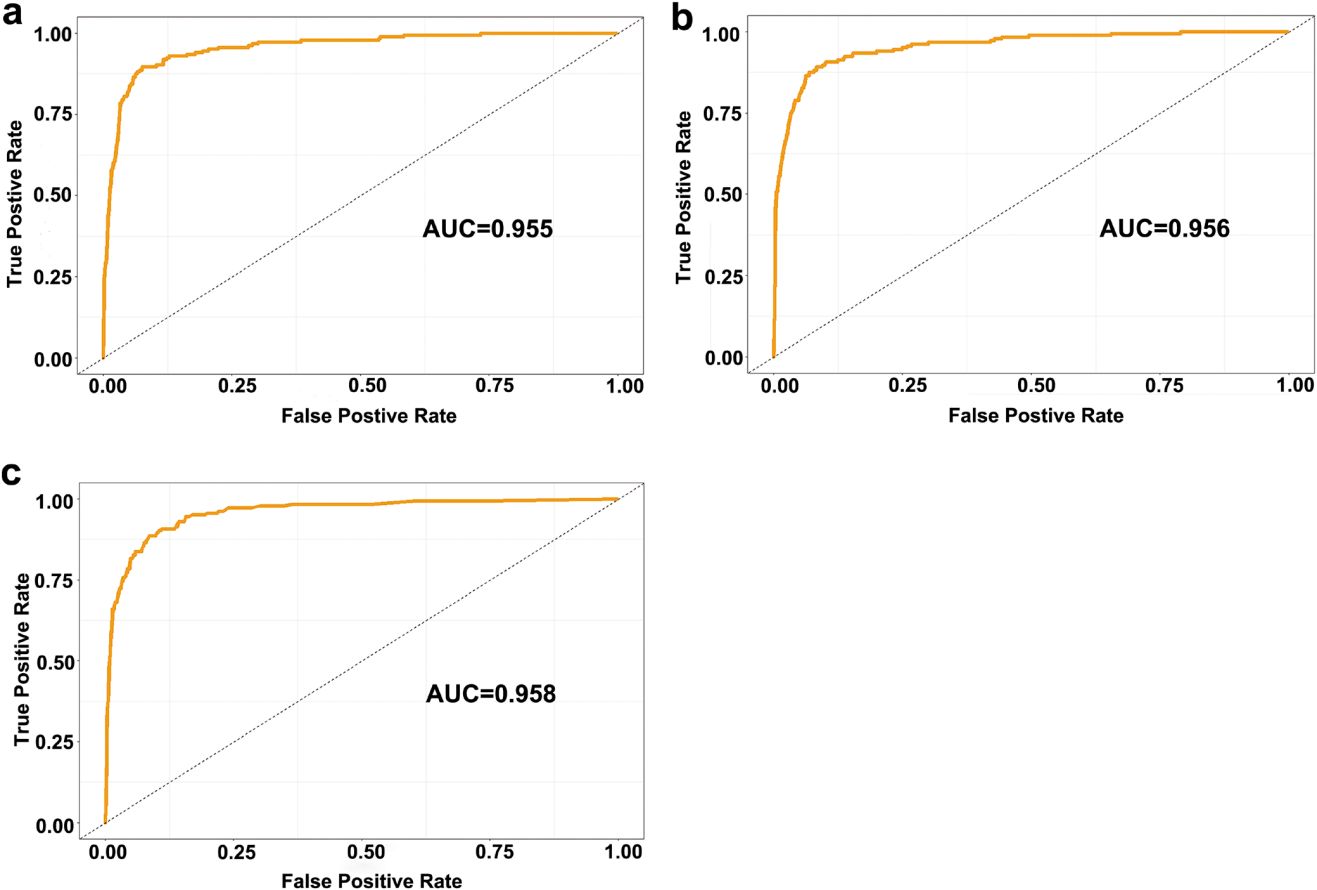
ROC curve of the test set obtained by the diabetes machine learning prediction model (a) MLP, (b) XGBoost, and (c) RF.

**FIGURE 4:**
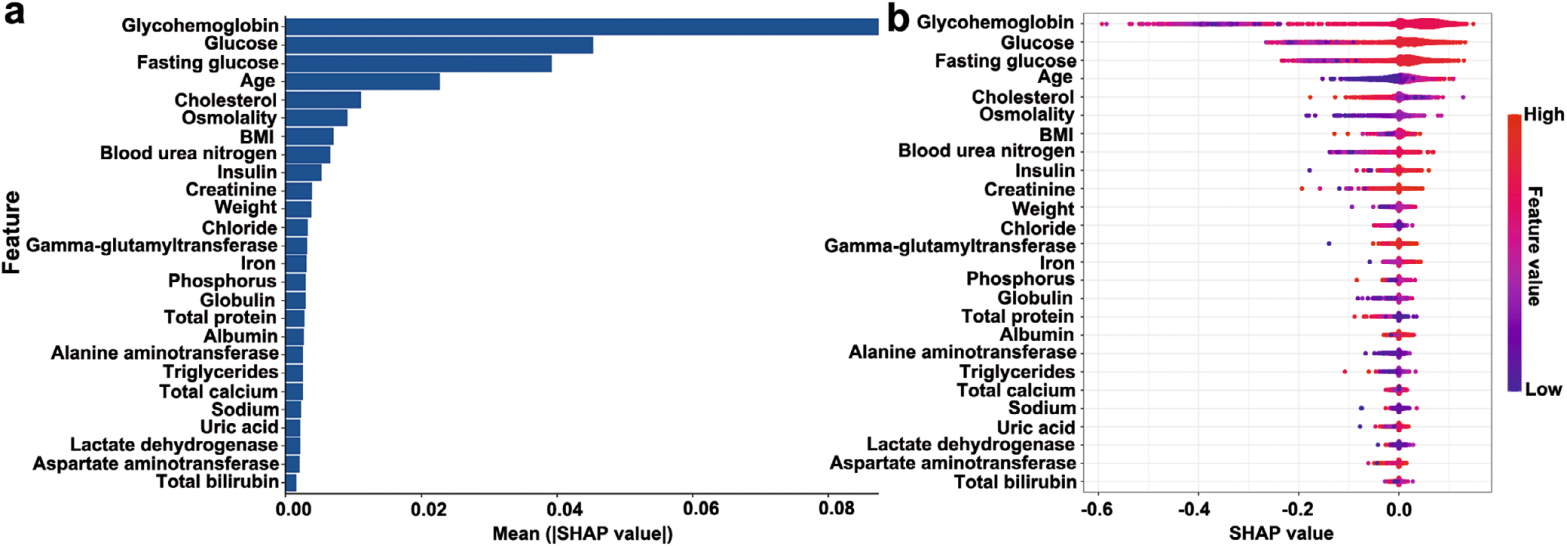
Model interpretation using the SHAP method. (a) The ranking of feature contribution to the RF model based on mean SHAP values. (b) The SHAP values were used to evaluate the impact of each feature on the prediction of diabetes by the RF model. Each dot in the figure represents a study participant. Dots are stacked vertically to show density. The color represents the value of the feature. The *x*‐axis represents the SHAP value. The more dispersed the dots of the graph represent the greater the impact of the feature on the model.

**FIGURE 5:**
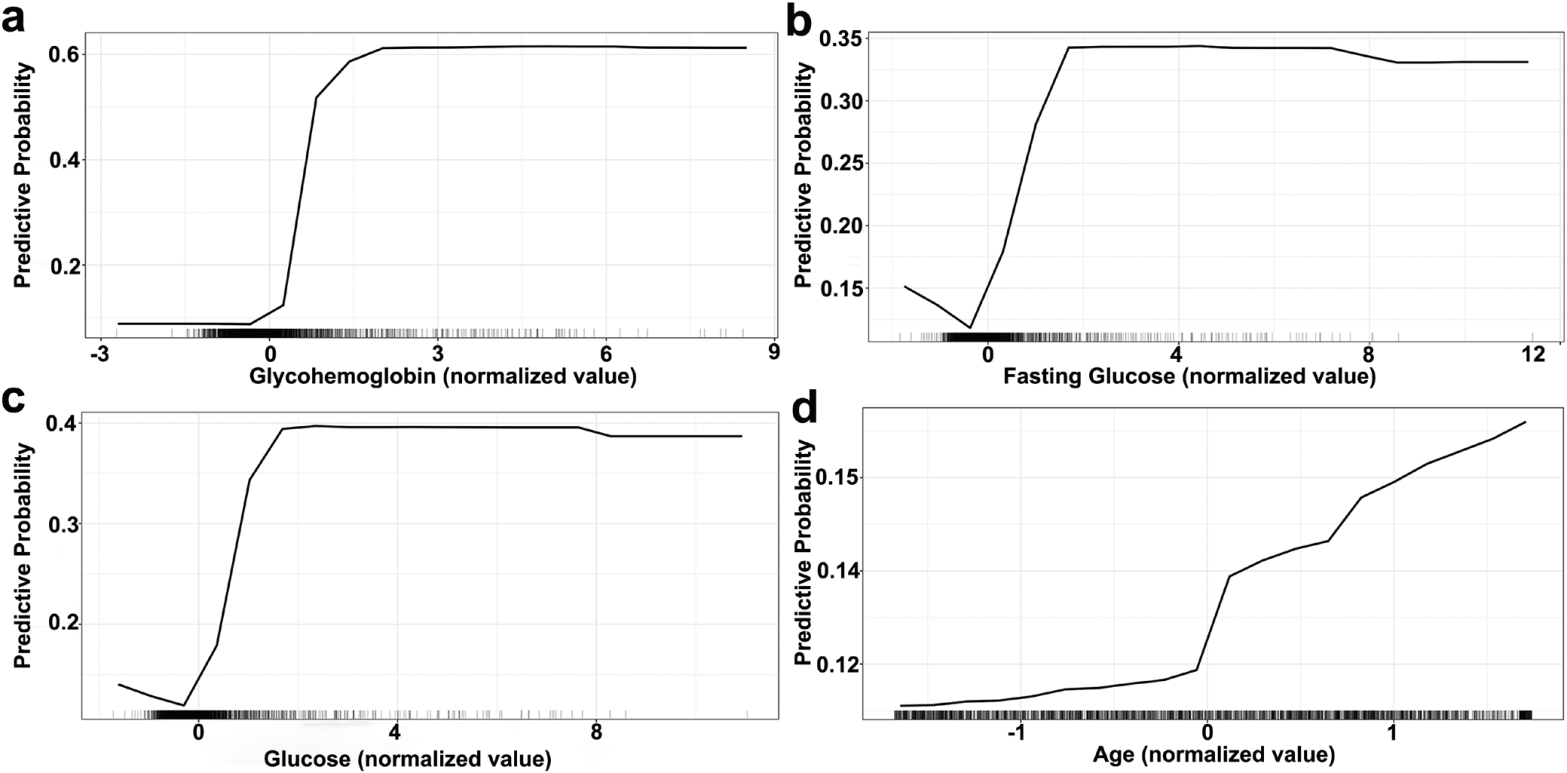
Partial dependency plot for the four features (a) glycohemoglobin, (b) fasting glucose, (c) glucose, and (d) age with the largest marginal contribution.

Therefore, SHAP and PDP were used in this study to analyze the impact of each feature in the RF model on diabetes diagnosis, and these results were shown in Figures 3 and 4. According to the SHAP values, the nine features that most contributed to diagnosing diabetes were glycohemoglobin (0.086), glucose (0.045), fasting glucose (0.039), age (0.023), cholesterol (0.011), osmolality (0.009), BMI (0.007), blood urea nitrogen (0.006), and insulin (0.005) (Figure 3a,b). Next, we selected the four features with the largest marginal contribution to explore how changes in features affect the model's diagnosis. According to the PDP plots, the findings indicated that the likelihood of developing diabetes increased in conjunction with elevated glycohemoglobin levels (Figure 4a). Furthermore, elevated glucose levels, high fasting glucose values, and older age also had a higher risk of developing diabetes (Figures 4b–d and 5). The above nine features can help identify people with a high risk of diabetes, which will help medical institutions and public health departments intervene in advance and reduce the incidence of diabetes through health education, lifestyle adjustments, and other means.

## 4 Discussion

In this study, the physiological characteristics and biochemical indicators from the population cohort in the NHANES database in the United States, spanning 2017 to 2020, were collected, and three machine learning approaches: RF, MLP, and XGBoost, were employed to develop diagnostic models for identifying diabetic patients. Among these models, the RF model exhibited the best performance, with an AUC of 0.958 (95% CI: 0.943–0.973), a recall of 0.897, specificity and F1 score of 0.916 and 0.747, respectively, and an overall accuracy of 0.913. Then, the RF diagnostic model was selected for model interpretation to further explore the risk factors affecting diabetes. Glycohemoglobin, glucose, fasting glucose, age, cholesterol, osmolality, BMI, blood urea nitrogen, and insulin exert the greatest influence on diabetes diagnosis. Thus, the RF model has considerable application prospects for the identification of diabetes and can serve as a valuable supplementary tool for clinical diagnosis and risk assessment in diabetes.

Diabetes has the characteristics of high morbidity and mortality. Early diagnosis can lead to timely treatment, thus effectively reducing the incidence and mortality of complications. Therefore, machine learning algorithms were used to construct diabetes diagnostic models by researchers to achieve early identification and intervention of the disease (Kopitar et al. 2020; Birjais et al. 2019; Basu and Narayanaswamy 2019). The RF algorithm was employed for diabetes diagnosis, with an accuracy of 0.906, a recall rate of 0.685 (Basu and Narayanaswamy 2019); the diabetes diagnosis model constructed by the CatBoost gradient boosting algorithm had an AUC of 0.82 (Kumar et al. 2022). The diabetes diagnosis model constructed using the XGBoost algorithm had an AUC of 0.9 (Lugner et al. 2024). The diagnostic performance of the RF model in our study was superior to previous studies, with an AUC of 0.958 (95% CI: 0.943–0.973), a recall rate of 0.897, and an accuracy of 0.913. This may be because we adopted a more sophisticated feature selection and cleaning method in the data preprocessing stage, which effectively reduced the interference of irrelevant data on model training, enabling the model to better fit the data characteristics, thereby showing a significant improvement in diagnostic performance.

Machine learning algorithm models usually have low interpretability (Li et al. 2023). So, we used SHAP and PDP as tools to enhance the interpretability of models, thereby gaining a deeper understanding of the basis for model diagnosis and further revealing the key factors of disease risk. This will not only increase transparency into machine learning predictions, but also provide clinical insights to help healthcare practitioners understand and prioritize risk factors (Tu et al. 2024). We found that the factors with the greatest impact on the prevalence of diabetes were glycohemoglobin, glucose, fasting blood glucose, age, cholesterol, osmotic pressure, BMI, blood urea nitrogen, and insulin. Consistent with our findings, Chowdhury et al. found that glycohemoglobin levels can be used as a diagnostic indicator for diabetes, and the higher the level of glycohemoglobin, the greater the risk of diabetes (Chowdhury et al. 2024). Fasting glucose levels and variables such as age, parental diabetes history, and BMI were also important factors in diagnosing diabetes (Lv et al. 2023; Wilson et al. 2007). In addition, a comprehensive literature review revealed that the main factors affecting the risk of diabetes were as follows: age, gender, height, BMI, waist circumference, blood pressure, and high‐density lipoprotein cholesterol (Lega and Lipscombe 2020; Sharma and Shah 2021). SHAP analysis in this study showed that blood urea nitrogen and osmotic pressure are important risk factors for predicting diabetes, but there are relatively few studies on their impact on the pathogenesis of diabetes.

In conclusion, the RF model constructed in this study was accurate and robust in diagnosing diabetic patients and can serve as a valuable supplementary tool for clinical diagnosis and risk assessment of diabetes. Clinicians can use the RF model to diagnose individuals who are at high risk of diabetes and conduct lifestyle intervention or drug treatment for them in advance to reduce the morbidity and mortality of diabetes. However, this study has certain limitations. Firstly, the information of the participants was from questionnaires in the NHANES database, which may introduce errors due to recall bias. These errors may affect the accuracy of the machine learning model in identifying diabetes. Secondly, this is a single‐center study, and the constructed machine learning model has not been verified by external data. External validation can determine whether the performance of the predictive model under different independent data sets is accurate and robust. To verify the universality and generalization ability of the model, subsequent research was needed to use database data from multiple sources for multi‐center verification.

## Author Contributions

**Zhihui Xiao:** conceptualization (equal), data curation (equal), formal analysis (equal), investigation (equal), methodology (equal), writing – original draft (equal). **Mingfu Wang:** supervision (equal), validation (equal), writing – review and editing (equal). **Yueliang Zhao:** conceptualization (equal), supervision (equal), validation (equal), writing – review and editing (equal). **Hui Wang:** supervision (equal), validation (equal), writing – review and editing (equal).

## Ethics Statement

No ethical approval or consent was necessary for this study, as it used publicly accessible data. The study population data are from the NHANES database (https://wwwn.cdc.gov/nchs/nhanes).

## Conflicts of Interest

The authors declare no conflicts of interest.

## Data Availability

The data that support the findings of this study are available on request from the corresponding authors.
